# Expanded consumer niche widths may signal an early response to spatial protection

**DOI:** 10.1371/journal.pone.0223748

**Published:** 2019-10-15

**Authors:** Angeleen M. Olson, Rowan Trebilco, Anne K. Salomon

**Affiliations:** 1 School of Resource and Environmental Management, Simon Fraser University, Faculty of Science, Burnaby, British Columbia, Canada; 2 Hakai Institute, Heriot Bay, British Columbia, Canada; 3 Antarctic Climate and Ecosystems CRC, University of Tasmania, Hobart, Tasmania, Australia; Australian Bureau of Agricultural and Resource Economics and Sciences, AUSTRALIA

## Abstract

Marine management interventions are increasingly being implemented with the explicit goal of rebuilding ocean ecosystems, but early responses may begin with alterations in ecological interactions preceding detectable changes in population-level characteristics. To establish a baseline from which to monitor the effects of spatial protection on reef fish trophic ecology and track future ecosystem-level changes, we quantified temperate reef fish densities, size, biomass, diets and isotopic signatures at nine sites nested within two fished and one five-year old marine protected area (MPA) on the northwest coast of Canada. We calculated rockfish (Sebastes spp.) community and species-specific niche breadth for fished and protected areas based on δ^13^C and δ^15^N values. We found that rockfish community niche width was greater inside the MPA relative to adjacent fished reefs due to an expanded nitrogen range, possibly reflecting early changes in trophic interactions following five years of spatial protection. Our data also demonstrated that the MPA had a positive effect on the δ^15^N signature of rockfish (i.e., trophic position), but the effect of rockfish length on its own was not well-supported. In addition, we found a positive interaction between rockfish length and δ^15^N signature, such that δ^15^N signatures of rockfish caught within the MPA increased more rapidly with body size than those caught in fished areas. Differences in rockfish size structure and biomass among fished and unfished areas were not clearly evident. Species of rockfish and lingcod varied in trophic and size responses, indicating that life-history traits play an important role in predicting MPA effects. These results may suggest early changes in trophic behavior of slow-growing rockfish due to predation risk by faster growing higher trophic level predators such as lingcod inside MPAs established on temperate reefs. Consequently, spatial protection may restore both the trophic and behavioral roles of previously fished consumers earlier and in measurable ways sooner than observable changes in abundance and size.

## Introduction

Despite increasing calls and legislated mandates for ecosystem-based management (EBM) of terrestrial and marine systems worldwide [1–4], applied marine ecology and fisheries conservation science tends to focus on changes in the population abundance or biomass of key species, rather than changes in the trophic and behavioural interactions that underpin ecosystem-level processes. This tendency is borne out of necessity, because it is much harder to observe and measure species interactions than it is to count and estimate individuals within a population. Yet, system change often begins with alterations to flows (e.g., trophic rates), with detectable changes in stocks (e.g., population abundances) lagging behind [5]. In marine systems, where the tenets of EBM now underlie efforts to rebuild entire marine ecosystems rather than their individual parts [6–8], great opportunities exist to develop and apply tools that can detect the effects of management interventions on species interactions underlying change in ecosystem structure and function, even before changes in population abundances are detectable.

The sum of the interactions that link species in an ecosystem is captured by the niche concept [9,10]. While the niche concept has evolved, contemporary definitions retain the formalization of the niche as a multidimensional space capturing both resource and habitat use [11]. Stable isotopes provide one increasingly widely-used empirical approach for describing niche dimensions [12]. By integrating species interactions over time, stable isotopes can quantitatively characterize community-wide aspects of trophic structure and diversity at the level of an entire food web [13]. Because species and community isotopic niches are an emergent outcome of the species interactions and energy flows that underlie ecosystem structure and function, alteration to these niches may signal ecosystem-wide effects of management interventions.

Marine protected areas (MPAs) are a form of EBM intervention in which changes in species interactions have been predicted theoretically [1,14,15] and observed empirically [15,16]. In many cases, the mechanisms driving these changes involve the recovery of previously fished top predator abundance and size structure, leading to higher predation rates on prey, and in some cases trophic cascades with wide ranging ecosystem-level consequences [17–20]. Changes in food web interactions associated with predator recovery can be detected by examining the alteration of a predator's trophic niche [21]. Yet, the occurrence and magnitude of these management intervention effects on system-wide interactions are highly context-dependent [20,22], varying with ecosystem diversity, regional oceanography, habitat complexity, and the nature and magnitude of the fisheries involved [23]. Furthermore, fishery-induced alterations to species interactions typically exhibit time lags, with differential response times varying among trophic levels [23,24] and life-history traits [25], with long-lived, late-to-mature fish typically predicted to exhibit time lags in behavioral and numerical response to protection.

Surprisingly, however, empirical evidence from multiple MPAs have revealed relatively rapid manifestations of direct effects on target species and lagged indirect responses by non-target taxa. In a meta-analysis of tropical and temperate protected and fished reef habitats, Babcock et al. [24] detected direct reserve effects on target species within 5.13 ± 1.9 years, an unexpected result given the life-history characteristics of most targeted species being long-lived and relatively slow growing. Colonization through cross-boundary movement of mature adult individuals was postulated as one plausible explanation, which has been demonstrated empirically [26,27]. Furthermore, indirect effects on non-target taxa took significantly longer to manifest (13.1 ± 2.0 years), in part due to behavioral modifications of lower trophic level prey that reduced their risk of predation [24]. These trait-mediated effects on species recovery times and variable recovery rates across trophic levels within both rocky and coral reef ecosystems suggest that many of the ecological processes we aim to conserve with MPAs operate on multiple and sometimes unexpected time scales.

In nearshore temperate rocky reef ecosystems along the northeastern Pacific coast, lingcod (*Ophiodon elongatus*), a dominant reef predator, and rockfish (*Sebastes* spp.), long-lived reef mesopredators, have experienced significant declines in abundances and size due to commercial and recreational overfishing [28–30]. In an effort to reverse declines, rockfish conservation areas (RCAs), a type of MPA closed to hook-and-line fishing, were designated along the west coast of Canada and the United States in the early 2000s [30,31]. Rockfish species are long-lived, late-to-mature mid-trophic level predators that can exhibit slow responses to spatial protection. Lingcod on the other hand, are faster growing, higher trophic level predators that appear to respond more quickly to spatial protection [32–34]. Because RCAs are protecting rockfish and, indirectly, their predators, lingcod, previous theoretical and empirical research suggests that the dynamics of predator-prey interactions could be changing in multiple ways within these MPAs [14,32,35]. Understanding how management interventions, like spatial protection, affect trophic interactions is important given the multiple, and at times competing, EBM objectives associated with them [36], in this case to recover rockfish, lingcod, and restore ecosystem processes.

**Fig 1:**
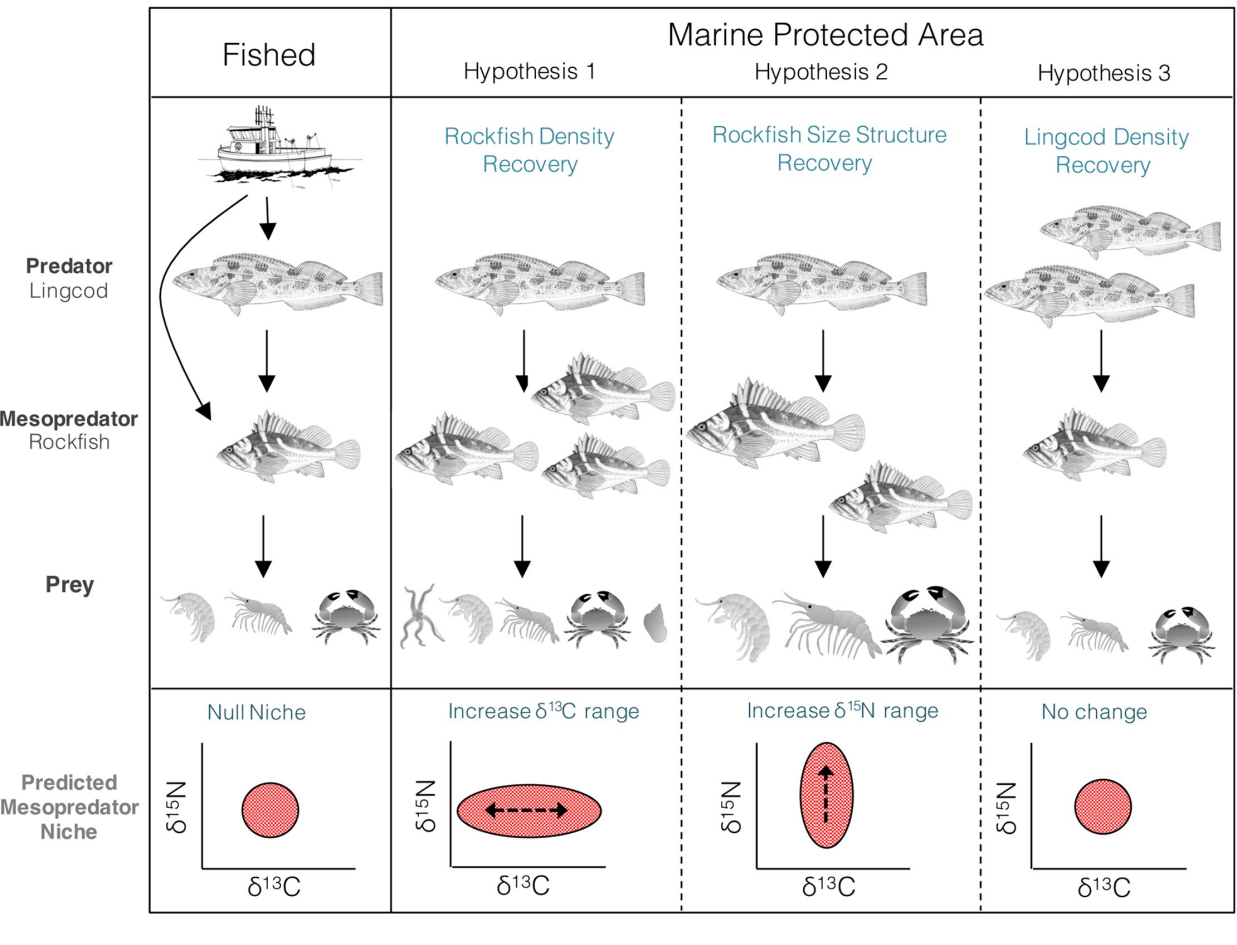
Hypothesized trophic niche widths of rockfish communities in response to changes in their density, size, and trophic interactions in an MPA compared to fished areas. The first panel depicts a simplified temperate reef food web in which lingcod are dominant predators of rockfish, mesopredators that primarily consume benthic invertebrates and fish. A hypothetical community isotopic niche space is depicted as a red circle. Hypothesis 1 predicts that the cessation of fishing rockfish within an MPA will lead to increases in rockfish density, prompting competition among mesopredators, and the expansion of their trophic niche width in the δ^13^C range. Hypothesis 2 predicts an expansion in rockfish size structure, thereby expanding rockfish trophic niches in the δ^15^N range. Hypothesis 3 illustrates a faster recovery of piscivorous lingcod in the MPA that slows rockfish recovery, resulting in no expansion of rockfish trophic niche width.

Here, we 1) establish a baseline of reef fish trophic position and ecology within a five year-old MPA designated specifically to support rockfish recovery and adjacent fished areas comparable in dominant biophysical characteristics, from which future ecosystem-level changes can be tracked; and 2) assess the evidence for initial differences in reef-associated species interactions, trophic position and rockfish community niche width among these areas. To do so, we examined the relative strength of evidence for three alternative, non-mutually exclusive hypotheses regarding changes in rockfish community niche width as a result of MPA establishment (Fig 1).

We hypothesized that reduced fishing pressure in MPAs can cause an increase in rockfish density, thereby increasing competition among individuals and prompting a diversification of prey (and thus basal resources) in rockfish diets relative to fished areas. An expansion in the δ^13^C range of the rockfish community trophic niche width would reflect this response (Hypothesis 1). Alternatively, a release from size-selective fishing pressure within MPAs may drive a change in size structure by increasing the relative abundance of larger individuals. This expansion of rockfish trophic diversity would be reflected by an expansion of rockfish niche width in the δ^15^N range (Hypothesis 2). Lastly, predatory lingcod may also increase in density and size in response to spatial protection. Increased lingcod predation may offset MPA-induced rockfish population changes, resulting in no differences in rockfish community niche relative to fished areas (Hypothesis 3). To test these hypotheses, we quantified and compared rockfish community and species-specific niche widths between replicate sites nested within a five year-old MPA and adjacent fished areas with similar biophysical characteristics. We took a model selection approach to quantify the strength of evidence for alternative factors driving differences in rockfish trophic ecology, size, density, and biomass among areas.

## Materials and methods

### Study region and design

**Fig 2:**
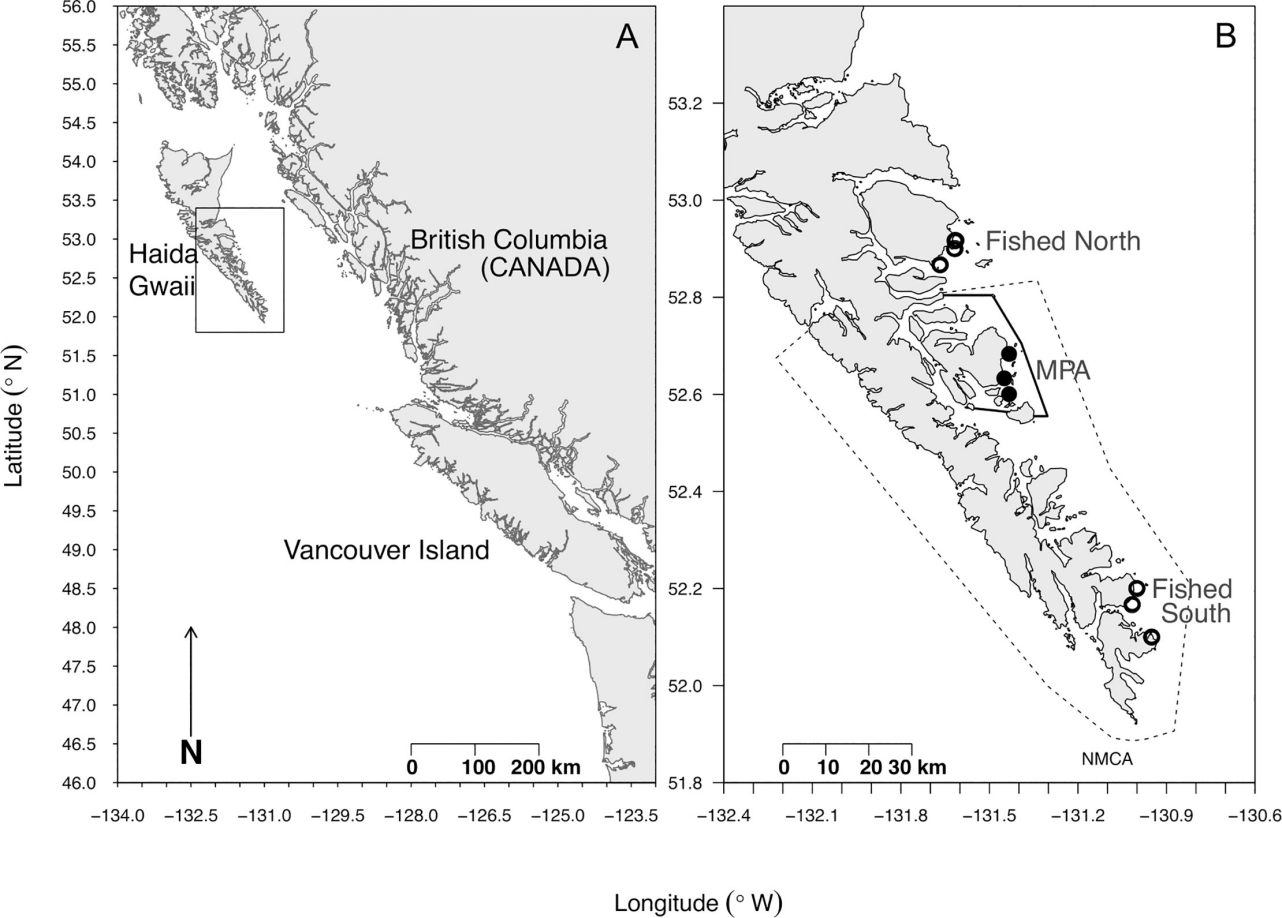
**This research was conducted in 2009 on shallow rocky reefs surrounding (A) Haida Gwaii, an archipelago located off the northwest coast of British Columbia, Canada. (B) We surveyed nine sites nested within three areas; two areas open to hook-and-line fishing (open circles) and one area closed to hook-and-line fishing in 2004 (closed circles, solid line).** An NMCAR was established in 2010 (dashed lines) in which marine planning and ocean zoning is currently underway, but not implemented.

To establish a baseline for reef fish trophic ecology and assess the initial ecosystem-level effects of an MPA located off the east coast of Haida Gwaii, an island archipelago located in British Columbia, Canada (Fig 2), we surveyed reef fish and habitat characteristics at nine replicate rocky reef sites nested within two fished areas (Kunghit Island, ‘Fished North’, n = 3 sites; Louise Island, ‘Fished South’, n = 3 sites) and an MPA (Lyell Island, n = 3 sites) in July and August 2009. Within areas, sites ranged from 3.4 km– 13.3 km apart. Areas, and the replicate sites nested within them, were comparable in their habitat, substrate complexity (Figs A-B in S1 File), depth range, aspect, wave exposure, and regional oceanographic context [37], but differed in their exposure to fishing pressure (i.e., hook-and-line fishing is absent in the RCA and present at fished sites). At each site, we quantified reef fish density and sizes and collected rockfish tissue and gut samples for niche characterization. The MPA was established as an RCA in 2004, making it five-years old at the time of surveying. With this MPA designation, commercial and recreational hook-and-line fishing is prohibited, however several invertebrate and forage fish fisheries are permitted [30].

All sites were embedded within what is now the Gwaii Haanas National Marine Conservation Area Reserve (NMCAR) established in 2010 (Fig 2B), a year after our 2009 surveys. This large conservation area surrounds the land-based Gwaii Haanas National Park Reserve and Haida Heritage Site, extending spatial protection 10 km offshore, creating an integrated terrestrial and marine conservation area of ~5,000 km^2^ [38]. At the time of establishment, the NMCAR only prohibited offshore drilling, however future ocean zoning and no-take protected areas are currently being planned for this larger ~3,400 km^2^ NMCAR [38]. Our subtidal surveys were initiated so that baseline conditions prior to the implementation of this larger marine spatial plan with the NMCAR could contribute towards future ‘before- after- control-impact’ assessments of the marine plan implementation. Monitoring efforts are continuing in this area and future studies, like that of Babcock et al. [24], will assess decadal trends in these fished and protected sites. As of 2019, this RCA is being re-zoned within a new spatial plan [39].

Studying the ecosystem effects of management interventions, like MPAs, at ecological scales germane to management and conservation is often constrained by the number and spatial extent of the management interventions, a paucity of true controls, high degree of variability among sites owing to factors other than the intervention, and a lack of ‘before intervention’ data. Most studies designed to evaluate MPA effects are confronted with these challenges and it is the case here, where we have one MPA, no pre-establishment data, and fished sites that may differ from MPA sites in ways other than their exposure to fishing pressure. To overcome some of these common barriers, we used a nested, hierarchical survey design, with replicate sites nested within protected and fished areas, as has been done to test the MPA effects elsewhere [19,40,41]. We specifically chose fished sites with biophysical characteristics as similar as possible to those in the MPA. Moreover, the time since MPA establishment in our case was short (five years), especially relative to the life history traits of rockfish, which are long-lived, slow growing and slow to reach maturation [28]. The data presented here represent a snapshot at the beginning of a recovery trajectory, with a monitoring program designed with the long-term assessment of this MPA and NMCAR in mind [37].

### Reef fish and habitat sampling

To assess the density, biomass, and size of reef-associated fish, we conducted subtidal visual transects by scuba. At each site, we counted and estimated the length of all reef fish observed in 4 replicate 30 m x 4 m x 4 m belt transects placed at two depth ranges: shallow (5–8 m) and deep (10–13 m). Individual fish biomass was calculated using species-specific length-weight regressions [42]. Benthic reef habitat was classified categorically: gravel (0.5–5cm), cobble (5 cm– 30 cm), small boulder (30 cm– 1 m), medium boulder (1 m– 2 m), large boulder (> 2 m), or bedrock reef (>10 m). Classes were assigned an ordinal value denoting increasing habitat complexity. Kelp canopy cover was characterized categorized as zero, fragmented, or full kelp canopy.

We collected rockfish (n = 89) for stable isotope analysis using hook-and-line fishing methods at shallow (< 20 m), intermediate (20–33 m), and deep (> 33 m) depths using standardized lures. Fishing effort, specifically lure bottom time, was approximately equal across all sites. With approval from Simon Fraser University’s Animal Care Committee, fish were sacrificed immediately using a lethal dose (0.5 g / L) of MS222. Multiple species were caught and measured for weight and total length: *S*. *melanops* (black rockfish) n = 24, *S*. *pinniger* (canary rockfish) n = 5, *S*. *nebulosus* (china rockfish) n = 17, *S*. *caurinus* (copper rockfish) n = 26, *S*. *maliger* (quillback rockfish) n = 15, and *S*. *flavidus* (yellowtail rockfish) n = 2 (see Table A in S1 File for life-histories). White muscle tissue was dissected from the dorsal musculature behind the head and frozen immediately until laboratory processing. Fish stomachs were removed and dissected (n = 62 full stomachs). Functional groups of prey were assessed using mass and an index of relative importance (IRI) based on abundance, mass, and frequency of occurrence (for detailed methods see Appendix A in S2 File).

To assess the long-term diet assimilation of rockfish, we processed dorsal muscle tissue of rockfish for stable isotopes. Following standardized procedures for marine samples in Levin and Currin [43], frozen tissue samples were thawed and rinsed with 10% HCl, followed by de-ionized water baths to remove carbonates. Samples were dried at 60° C for 48 hours and ground to a powder and encapsulated for analysis at the UC Davis Stable Isotope Facility. δ^13^C and δ^15^N signatures were calculated relative to international standards, V-PBD for carbon and air for nitrogen:

$$\delta X=[\left(\frac{R_{sample}}{R_{standard}}-1\right)\;x\;1000)]$$ Eq 1

where X = ^13^C or ^15^N and R = ^13^C/^12^C or ^15^N/^14^N, respectively. δ units are in parts per thousand (‰), representing the relative enrichment of heavy to light isotope. Increasingly positive δ values represent increased enrichment of the heavy isotope. Accuracy is denoted as long-term standard deviation at UC Davis: 0.2 ‰ and 0.3 ‰ for δ^13^C and δ^15^N, respectively. We assumed that a shared isotopic baseline for δ^15^N was representative of all fish sampled in this study. This assumption was supported by the observation of a mean δ^15^N of 10.32 ‰ (n = 47, SD = 0.4) for rock scallops (*Crassodoma gigantea*) that were opportunistically collected at the same sites where fish were sampled (placing them one trophic level below the smallest fish sampled). Because they are among the longest-lived filter-feeding invertebrates present on these reefs, rock scallops provide a time-integrated baseline estimate for δ^15^N for this system. A summary of δ^15^N and δ^13^C for each fish species can be found in Table A in S2 File.

### Niche widths calculations and comparisons

To assess differences in rockfish niches, we first estimated isotopic niche widths for each species as multivariate standard ellipse areas (SEA) using methods developed by Jackson et al. [44] within the R package SIAR [45]. We estimated SEA using δ^13^C and δ^15^N means, variance, and covariance in a Bayesian framework and applied a correction factor to the bivariate data to prevent bias from small sample sizes, producing a final species niche estimate SEA_C_. SEA_C_ is robust to small samples sizes, as well as variable collection numbers [44]. Yellowtail rockfish were excluded from analyses due to limited sample size.

Rockfish community (e.g., species combined) niche breadth was estimated for each area. From Jackson et al. [44] methods, we estimated convex hulls by connecting species derived SEA_C_ estimates. This method incorporates natural propagation of uncertainty, and the ability for statistical comparison, building on initial community niche metrics proposed by Layman et al. [13]. From the convex hulls, we estimated four metrics that assess rockfish community niches that indicate various aspects of trophic diversity within the food web [44]: total area (TA), nitrogen range (NR), carbon range (CR), and mean distance to centroid (CD). TA is an estimate of total niche size, providing the total extent of trophic diversity within the community assemblage. NR and CR were calculated as the greatest distances between maximum and minimum δ^15^N and δ^13^C values, respectively, of the convex hull. NR represents the vertical structure of the food web and reflects the total number of trophic levels for within the community assessed, while CR provides an indicator of the diversity of basal energy inputs. CD, calculated by the distance between an individual and the δ^15^N - δ^13^C centroid in the community, provides a measure of mean trophic diversity.

### Data analysis

To determine if rockfish niche expansion occurred in the MPA (Fig 1), we compared niche metric isotopic extent between the MPA and each fished area, at the community level (niche metrics: TA, NR, CR, CD) and at the species level (niche metric: SEA_C_) among areas. We calculated the probability of niche expansion, by comparing the posterior distributions of the niche metrics (e.g., comprised of estimates of isotopic niche size) between areas, where the number of estimates greater in the MPA relative to a fished area, divided by the total number of estimates in the distribution, gives a probability of expansion [46]:

$$P\;(a>b)=\frac{\sum \left(X_{a}>X_{b}\right)}{n}\times 100$$ Eq 2

Where *a* and *b* represent the posterior distributions of a niche metric (e.g., TA) of differing areas (e.g., *a* = MPA and *b* = fished area). X represents a single estimate in the posterior distribution of metric *a* and *b*, and n is the total number of estimates in each posterior distribution (n = 10^4^). By estimating proportion of estimates in *a* that were larger than *b*, we calculated the direct probability of the niche expansion in the MPA relative to fished areas. We further extended these methods to compare niche sizes among different species (e.g., *a* = black rockfish vs. *b* = copper rockfish).

We constructed linear mixed effects models (LMEs) for rockfish communities, species-specific rockfish, and lingcod observed on scuba transects to test for the effects of spatial protection on fish densities, sizes, and biomass. Models of log transformed fish biomass were analyzed with a Gaussian error distribution and identity link function and fitted by residual restricted maximum likelihood (REML) using the R package lme4 package [47]. Generalized linear mixed effects models (GLMMs) of fish density were fit with a Poisson error distribution and log link function. Protection status (*status)*, kelp canopy cover *(kelp)*, depth, and benthic habitat class (*benthic habitat)* were treated as fixed factors and assigned an ordinal value of increasing values representing more protection, kelp canopy cover, depth, and rock size, respectively. *Site* was treated as a nested random effect due to the hierarchical nested nature of our sampling design [48]. Differences in community and species-level size structure were assessed with size-frequency distributions between fished areas and the MPA.

To determine the effects of spatial protection on rockfish δ^15^N signatures (e.g., trophic diversity), we used LMMs. Protection *status* was treated as a fixed-effect, as well as rockfish body size (*length)* because size is known to effect δ^15^N signatures, a proxy of trophic level [49]. We included an interaction between *length* and *MPA status* to test if protection status mediated the relationship between rockfish size and δ^15^N. In this model, *site* was included as a random effect. In addition, we tested for separate effects of each fished areas (Fished North and Fished South) on the relationship between fish length and δ^15^N by treating *area* as a fixed effect. This model showed no difference in the relationship between fish length and δ^15^N among fished areas (Table A and Fig A in S3 File).

We used a model selection approach to evaluate the strength of evidence for alternative candidate models of reef fish biomass, density, and rockfish δ^15^N values. We compared models with combinations of fixed factors that we deemed ecologically relevant *a priori*. The relative support for each model was determined using Akaike information criterion (AIC) corrected for small sample size (AICc). Models were standardized relative to the best-fit and most parsimonious model to determine ΔAICc values, where lower values indicate the best compromise between model fit and complexity. ΔAICc values of ≤ 2 were used as a cut-off to separate well-supported models from those that were less well-supported [50]. Akaike weights (W_i_) were calculated as the ratio of Δ_i_ values for each model relative to the entire set of candidate models. To assess the relative importance of each variable in our biomass and density model sets, we averaged the top models using Akaike weights in a weighted average. Using the R package *MuMIn* [51], average parameter estimates and unconditional variance were calculated from models with ΔAICc values < 4, as well as their relative variable importance (RVIs), the sum of the Akaike weights of the models in which that factor appears. Prior to model averaging, we standardized the continuous predictor variables by subtracting their mean and dividing by 2 standard deviations for direct comparison of parameter coefficients [52].

## Results

### Differences in rockfish community & species niches between the MPA and fished areas

**Fig 3:**
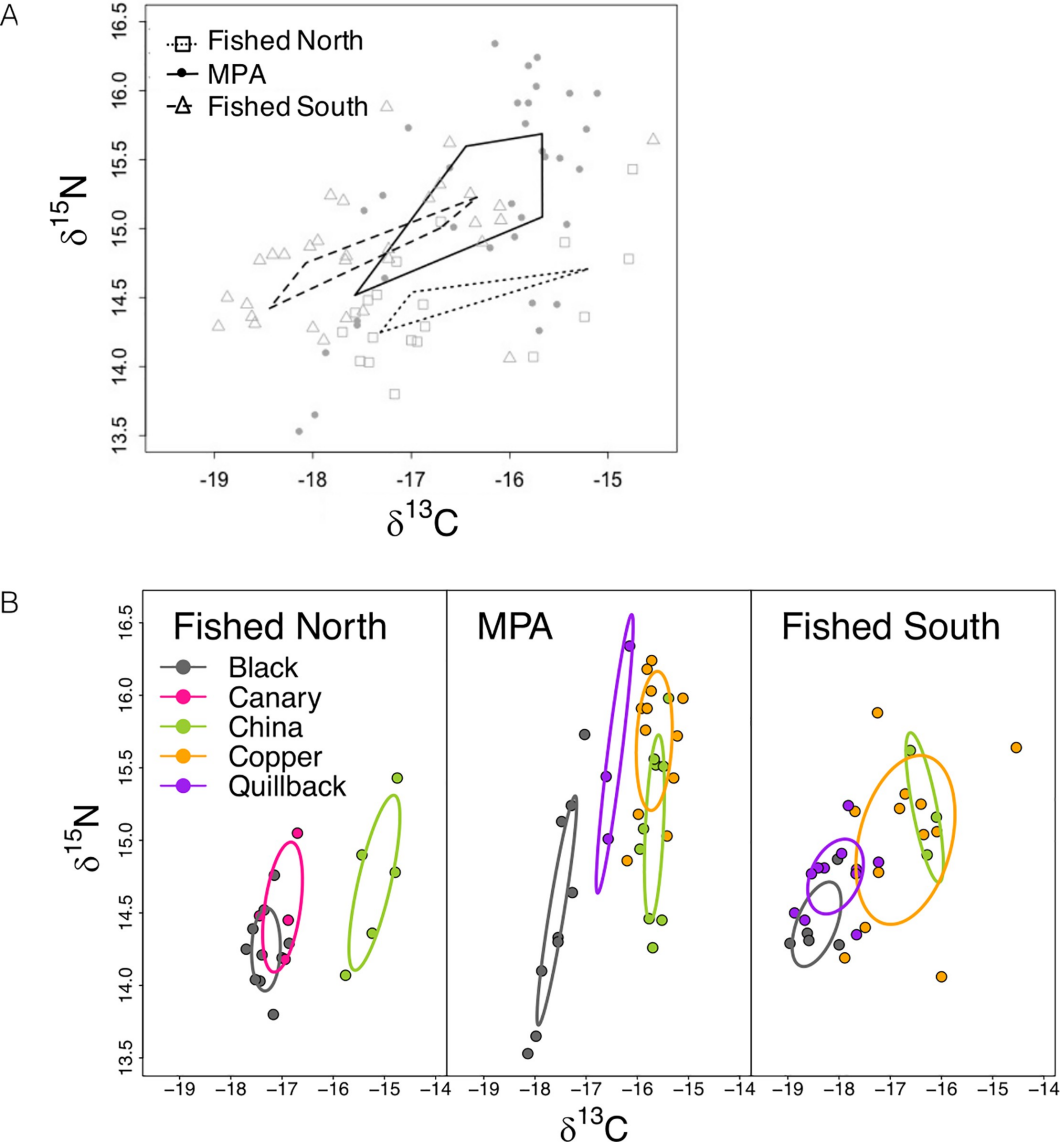
Rockfish isotopic niches. (A) Community niche widths among sites nested within two areas open to fishing (open symbols and dashed lines) and protected sites closed to fishing in an MPA (closed circles and solid lines). (B) Species-specific niche widths (SEA_C_) across Fished North, the MPA and Fished South: black rockfish (grey), canary rockfish (pink), china rockfish (green), copper rockfish (orange), and quillback rockfish (purple).

We found an 86% and 72% probability that the total extent of rockfish community trophic diversity, indicated by total community niche space (TA), was larger in the MPA than Fished North and Fished South, respectively (Fig 3A; Table 1). Niche expansion in the MPA was largely due to an increase in trophic level diversity indicated by a wider nitrogen range (NR extent). Specifically, the rockfish community NR within the MPA had an 89% probability of being larger than that of the Fished North and a 73% probability of being larger than that of the Fished South. There was a lower probability (39% Fished North; 37% Fished South) that the protected and fished areas’ rockfish community niches differed in their isotopic carbon range (CR).

**Table 1:** Community-wide measures of rockfish trophic structure compared between the MPA and each fished area (North and South). Total area (TA) of the convex hulls represents the total niche space occupied and thus total extent of trophic diversity in the food web. Nitrogen range (NR) represents the vertical structure within a food web, and carbon range (CR) represents the range in basal resources at the base of a food web. Mean distance to centroid (CD) provides an average measure of trophic diversity and is a function of species spacing. Probability (%) of difference in niche metrics were calculated with *Eq 2*.

Rockfish Community Niche Measure of Trophic Structure	Probability (%) of Niche Metric Difference		
	MPA > Fished North	MPA > Fished South	Fished South > Fished North
Total Extent of Trophic Diversity (TA)	86	72	71
Trophic Level Diversity (NR)	89	73	70
Basal Resource Diversity (CR)	39	37	53
Trophic Diversity Species Spacing (CD)	45	42	53

Rockfish species-specific niches (SEA_c_) differed between fished and protected areas for some species, but not all (Fig 3B; Table 2A). Black rockfish had an 87% probability of having a larger niche width at sites within the MPA relative to Fished North, yet only a 44% probability of having a larger niche compared to Fished South (Fig 3B; Table 2A). We found very low probabilities (17–34%) that china and copper rockfish niche widths differed between the MPA sites and the sites nested within both fished areas. Quillback rockfish had a 93% probability of having a larger niche width in the MPA than Fished South. No quillback or copper rockfish were caught in the fished area north of the MPA and were unable to compare to those sites.

**Table 2:** Species-specific trophic niche comparisons. Rockfish species SEA_C_, a measure of isotopic niche width, compared (A) among areas and (B) within areas. Probability (%) of difference in species niche width were calculated using *Eq 2*.

**A) Species**	**Area Comparison**		**% Probability of Niche Expansion**
Black rockfish	MPA >	Fished North	87
	MPA >	Fished South	44
	Fished North >	Fished South	12
China rockfish	MPA >	Fished North	22
	MPA >	Fished South	17
	Fished North >	Fished South	39
Copper rockfish	MPA >	Fished South	4
Quillback rockfish	MPA >	Fished South	93
**B) Area**	**Species Comparison**		**% Probability of** **Niche Expansion**
Fished North	Canary >	Black	92
	China >	Black	92
	China >	Canary	49
MPA	Quillback >	Copper	95
	Quillback >	China	89
	Quillback >	Black	81
	Copper >	Black	20
	Copper >	China	34
	China >	Black	33
Fished South	Copper >	Quillback	95
	China >	Quillback	90
	Black >	Quillback	79
	Copper >	Black	69
	China >	Black	68
	China >	Copper	54

When species-specific niche widths (SEA_C_) were compared within areas, we found that species occupied unique niche spaces of varying position and size (Table 2B, Fig 3B). At Fished North, canary and china rockfish each have a 92% probability of having a greater niche width than black rockfish, while china and canary rockfish niche sizes did not differ from each other (Table 2B). Quillback rockfish niche width at the fished area south of the MPA had a 95%, 90%, and 79% probability of being smaller than copper, china, and black rockfish, respectively. Conversely, quillback rockfish niche width in the MPA had a 95%, 89%, 81% probability of being greater than copper, china and black rockfish niche widths, respectively. Across all areas, both fished and protected, china rockfish consistently tended to be enriched in both δ^15^N and δ^13^C compared to black rockfish.

### Factors driving an increase in δ^15^N range

**Fig 4:**
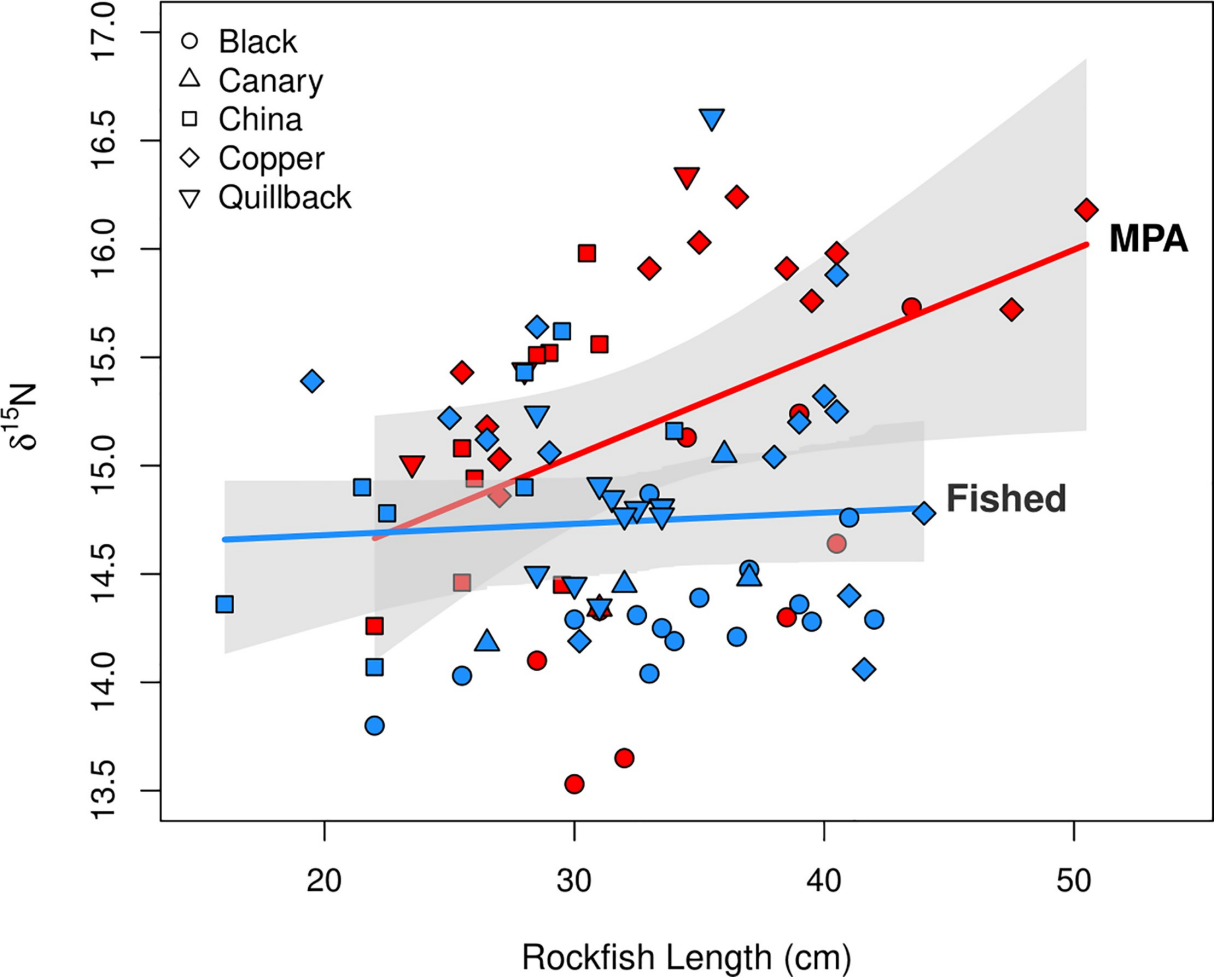
Community δ^15^N and body length relationships. Relationship between rockfish δ^15^N and body length, as a function of MPA protection status (*b* = 0.05, R^2^ = 0.19) with 95% confidence intervals (shaded bands).

We detected evidence that protection status had a positive influence on rockfish δ^15^N signature, a measure of trophic diversity, of fish inhabiting shallow reefs off Haida Gwaii (model W_i_ = 0.70, Table 3, Fig 4). Further, we found that protection status mediated the relationship between rockfish length and δ^15^N signature (Fig 4), such that δ^15^N signatures of rockfish caught within the MPA increased more rapidly with body size than those caught in fished areas (Fig 4). Copper, black, and, quillback rockfish species appear to be driving this trend. The potential positive effect of rockfish length, irrespective of protection status, was imprecise (confidence intervals cross 0). We did not find evidence for differences in effects of Fished North and South on the relationship between rockfish length and δ^15^N (Table A and Fig A in S3 File).

**Table 3:** Relative support of models explaining δ^15^N as a function of rockfish body length and protection status. Models were compared using likelihood of model given the data (Log(L)), K, and ΔAIC_C_ values. Asterisks (*) represent an interaction term. The top model is shaded grey.

Response	Model	K	Log(L)	AIC_c_	Δ AIC_c_	W_i_
Rockfish δ^15^N	Length + Status + Length*Status	6	-77.97	166.7	0.00	**0.70**
	Length + Status	5	-80.19	168.9	2.19	0.25
	Status	4	-82.79	171.9	5.19	0.05
	Length	4	-84.84	176.0	9.28	0.01
	Intercept	3	-87.34	178.8	12.15	0.00

### Stomach contents differences in rockfish between areas

Overall, rockfish diets in the MPA had high richness relative to the Fished North and South areas (Appendix A in S2 File). China, copper, and quillback rockfish species stomach contents reflected this pattern, while black rockfish displayed less rich diets. However, the composition of prey consumed by rockfish was variable between species and areas, as indicated by mass and IRI prey items. At Fished South, most species (china, copper, and quillback), consumed a high proportion of small cryptic crabs (e.g., *Pugettia richii*, *Scrya acutifrons*, *Cancer oregonensus*), or fish (e.g., black rockfish). At Fished North, rockfish exhibited species-specific differences in diet, however amphipods were most prevalent here compared to the MPA and Fished South. Species displayed unique foraging preferences, such that black rockfish diets were typically dominated by *Caprella alaskana*, fish, and zooplankton. China and copper rockfish diets showed high proportions of small cryptic crabs and quillback rockfish consumed crabs and amphipods. Diets of quillback and copper rockfish at Fished North were highly diverse.

### Rockfish density, biomass, and size in fished and protected areas

We did not clearly detect a greater density or biomass of the rockfish community at sites within the MPA compared to sites in adjacent fished areas based on underwater visual surveys (Fig B in S3 File). Rather, we observed high intra and inter-site variability, some of which was driven by schooling species (e.g., single, large school of black rockfish at a site within the northern fished area). We did not detect evidence for positive effects of protection status on any specific rockfish species biomass or density (Table B and Figs C-D in S3 File), although quillback rockfish and lingcod densities tended to be slightly higher, yet variable, in the MPA relative to the fished sites as suggested by Hypotheses 1 and 3. Consequently, protection status had a positive, albeit imprecise and relatively weak effect on quillback rockfish and lingcod density (RVI = 0.23, 0.44 respectively; Fig D in S3 File).

We found evidence that benthic habitat type and kelp cover were important factors driving rockfish community density (RVI = 1 and 1, respectively) and black rockfish density (RVI = 1 and 1 respectively; Fig D in S3 File). MPA status was less important, and in fact had a negative effect on community biomass (RVI = 0.75) and density (RVI = 0.59), and black rockfish density (RVI = 0.82) and biomass (RVI = 1) (Figs C-D in S3 File), likely driven by the school in Fished North. We also detected evidence that china rockfish density was strongly influenced by both benthic habitat type (RVI = 1) and depth (RVI = 0.91) in combination (ΔAICc = 0, W_i_ = 0.47). Lastly, the size distributions of rockfish, at the community or species level, from scuba observations did not notably differ between fished and protected areas (Fig E in S3 File). In general, most rockfish were observed at Fished North, while lingcod were observed exclusively in the MPA and Fished South.

## Discussion

Recent evidence from marine protected areas established on both tropical and temperate reefs points to unexpectedly rapid manifestations of direct effects on long-lived target species and behavioral modifications by lower trophic level species that reduced their risk of predation [24]. Here, we present baseline data on the trophic structure of rockfish in a recently established MPA and adjacent fished sites with evidence for differences in their community niche width, signaling early changes in trophic interactions within this newly protected rocky reef ecosystem. Rockfish community niche width was greater inside the MPA relative to adjacent fished areas (Fig 3A, Table 1). This greater niche width was driven by higher trophic level diversity reflected by a wider nitrogen range, theoretically consistent with the hypothesis that a release from size-selective fishing pressure can enable larger rockfish to consume larger, higher trophic level prey (Fig 1; Hypothesis 2). Accordingly, protection status had a positive effect on rockfish δ^15^N (Fig 4), and mediated the positive relationship between rockfish size and δ^15^N. Contrary to this hypothesis, we did not detect a strong effect of the MPA on observable rockfish size. We hypothesize that behavioral responses by rockfish to higher order predation risk imposed by lingcod may precede observable population-level responses by rockfish. Species-specific life-history traits help to explain these seemingly paradoxical results.

### MPAs and the expansion of trophic level diversity

By supporting the recovery of previously fished predators, MPAs can alter food web structure [53,54], key trophic rates [18,19,55], and consumer niche breadth [21]. We found evidence that spatial protection may expand the dietary niche breadth of an entire rockfish community by increasing its trophic level diversity relatively rapidly. Rockfish community niche expansion in the MPA was driven by shifts of some species to higher trophic positions. Multiple, non-mutually exclusive ecological mechanisms could have triggered this response.

Our results suggest that recent spatial protection may alter rockfish feeding behavior. The δ^15^N signature of protected rockfish increased more rapidly with body size than those in fished areas, such that fish of equivalent size, and species composition, were more enriched in δ^15^N within the MPA (Fig 4). Given these trends and no detectable increase in the mean length or size distribution of rockfish communities in the MPA (Figs C-E and Table 2 in S3 File), our results imply protected rockfish were either feeding on different prey, or on similar prey that were at a higher trophic level. Observations of higher prey species richness in rockfish stomach contents from the MPA suggest differences in foraging compared to fished areas (Appendix A in S2 File). Dell et al. [56] similarly found that mesopredatory fish in MPAs foraged at higher trophic levels relative to fished areas despite smaller fish sizes in the MPA. This was caused by a prey switch from invertebrates to small recruiting fish that were more abundant in the MPA than non-MPA areas. In contrast, kelp bass (*Paralabrax clathratus*) in southern California MPAs did not demonstrate higher trophic positions than those at non-MPA sites, possibility due to ephemeral availability of pelagic food sources [57].

Previous studies on similar temperate reefs demonstrate strong behavioral responses by rockfish to predation risk from lingcod [58,59]. Lingcod are fast growing predators on temperate reefs known to consume rockfish [60] and respond positively to spatial protection [61]. We found that lingcod densities tended to be slightly higher, yet variable, in the MPA we surveyed relative to the fished sites (Fig B in S3 File). Empirical evidence from southern British Columbia suggests that lingcod have higher densities of larger spawning individuals in old, well-established MPAs relative to fished areas [62], which may have direct negative effects on sub-adult rockfish [59]. In fact, rockfish biomass consumed by lingcod in Washington State was 5–10 x higher in MPAs than adjacent fished areas [63]. Simulation models of lingcod and rockfish interactions within MPAs off the northeastern Pacific [64] highlight the generality of the community-level consequences of protecting apex predators on the recovery of their prey [1], although behavioral evidence would be needed to substantiate this hypothesis.

Rockfish display anti-predator behaviors in the presence of predators, such as avoidance and reduced attack rates on prey [58]. These behaviors are prevalent among slower-growing and late-to-mature rockfish (e.g., quillback and copper rockfish) relative to rockfish with faster life-history traits in similar trophic positions (e.g., black rockfish). Relative to Fished South, quillback rockfish niches had a 93% probability of niche expansion in the MPA (Fig 3B). In addition, we observed enriched δ^15^N signatures in quillback and copper rockfish in the MPA relative to Fished South, which suggests a behavioural change in foraging to higher trophic levels possibly to avoid lingcod predation (Figs 3B and 4A). Black rockfish also showed an 87% probability in niche expansion in the δ^15^N range in the MPA relative to Fished North, however less support for this was detected among this pelagic rockfish in Fished South (44% probability). Higher lingcod densities in the MPA relative to Fished North, but less so relative to Fished South (Figs B and D in S3 File), may have triggered behavioral shifts in rockfish foraging detectable in their isotopic signature.

Behaviorally-mediated interactions are well-documented among apex predators and mesopredators, in efforts by mesopredators to reduce the risk of predation [65]. Due to a trade-off with predation risk, foraging behaviors can be altered [66] where niches expansions to a more diverse set of prey occurs [67]. Given the unique species-specific life-history traits among reef consumers in our study, Hypotheses 1–3 (Fig 1) are not only non-mutually exclusive, but are likely co-occurring at different rates as the recovery of trophic and behavioral interactions plays out within this newly protected food web.

Studies elsewhere suggest that the recovery of apex predator size structure in MPAs can lead to an increase in food chain length (i.e. the number trophic levels) within a food web, because larger fish (i.e., with larger gape sizes) can consume larger, higher trophic level prey. This same mechanism was proposed for the dietary niche expansion of the California sheephead wrasse (*Semicossphus pulcher*) upon recovery of its population size structure following a reduction in previously intense fishing [21]. South of our study area, along the west coast of Vancouver Island where sea otter (*Enhydra lutris*) populations have been recovering and triggering a trophic cascade, increases in this apex predator and associated kelp forest habitat have been associated with expanded rockfish trophic niches, attributed to an increase in fish prey availability [68]. Here, we show that the trophic positions of reef fish in a Haida Gwaii ecosystem increase with body mass, which agrees with recent research [69], and our results indicate a positive MPA effect on trophic position. However, we did not observe larger or more abundant rockfish within the MPA. This is not surprising given just five years of spatial protection for a notoriously slow-growing fish genera (generation times 6–22 years, Table A in S1 File).

Rockfish species will likely respond to spatial protection in different ways due to their unique life-histories (Table A in S1 File). Species composition may thus play a key role in determining community-level niche responses. Here, rockfish species occupied unique niches within areas, and had varying responses in the MPA relative to fished areas (Fig 3B, Table 2B). For instance, unlike findings on Vancouver Island during the recovery of *E*. *lutris* [68], we did not see black and copper rockfish responding similarly in niche size. Alterations to trophic interactions, such as recovering higher trophic level predators, are expected to induce complex ecosystem changes, and thus affect these species uniquely, influencing how community niches will respond.

Another alternative explanation for the larger rockfish niche width in the MPA compared to fished areas, as well as differences in species composition between fished areas, is a difference in the underlying prey base among the geographic areas, unrelated to protection status. Because of the low sample size, due to a high proportion of empty stomachs of fish caught, the snapshot data afforded by stomach content analysis is less powerful than the stable isotope samples, which provide a long-term, integrated picture of prey assimilation. We acknowledge that our sample size, constrained by permits and sampling time, is small (n = 87 individuals among 5 species) and thus, the degree to which our estimates are representative of communities is constrained. While isotopic indices are a convenient proxy of trophic patterns, they also suffer from bias, especially when small sample size leads to wide dispersion, and thus should be interpreted carefully [70]. These limitations should be kept in mind when interpreting our results.

Long-term tracking of the trophic ecology and abundance of reef-associated fish across these sites through time and in comparison to this baseline survey will be necessary to distinguish among these possible explanations. Studies on how MPAs may alter the trophic positions of recovering consumers indicate that trophic responses will likely depend on ecosystem structure (e.g., habitat type, predators and prey), species characteristics (e.g., generalist vs specialist consumers), and/or regional biotic and abiotic processes. While emerging isotopic evidence has demonstrated that MPAs may alter trophic dynamics [56], other studies have found no effects [71,72] relative to adjacent areas. Moreover, niche responses to protection remain unclear, where expansion can occur when community size structure recovers [21], or shrink relative to adjacent fished areas due to differences in prey availability [57].

### Restoring trophic and behavioral roles of predators with spatial protection

Food web responses to spatial protection are complex and will continue to change as previously fished apex and mesopredators grow in size and grow in population abundance [1,14]. Density-dependent effects will likely drive benthic predators to expand their home ranges outside MPAs, exposing themselves to fishing mortality and once again altering the trophic interactions within MPAs [16,73]. Habitat changes can also occur via trophic cascades within MPAs [17,74], altering available habitat known to be important for reef-associated fish [75]. As such, trophic responses to spatial protection will change through time in unpredictable ways yielding ecological surprises [23]. We offer a benchmark by which to assess long-term changes in trophic structure post MPA establishment.

Ecosystem-based interventions, such as MPAs, are increasingly being used to rebuild ocean ecosystems. Yet, ecosystem change often begins with alterations in ecological interactions preceding detectable changes in populations. Fortunately, contemporary tools allow us to detect changes in trophic interactions that can otherwise escape observation. The patterns we document here imply that among ecosystems subject to spatial protection, dynamic and sometimes surprising changes to system flows (i.e., trophic interactions) may be anticipated before detectable changes in system stocks (i.e., population abundance and size structure). If the conservation objective of an EBM intervention is to restore ocean ecosystems, restoring both the trophic and behavioral roles of previously fished predators may manifest in measurable ways other than simply abundance and size.

## Supporting information

S1 File Habitat and fish life-history information. (DOCX)

S2 File Stomach content analysis and isotopic summaries. (DOCX)

S3 File Detailed model results. (DOCX)
