# Supplementary material for: Expanded consumer niche widths may signal an early response to spatial protection
Source: PLoS One. 2019 Oct 15;14(10):e0223748. doi: 10.1371/journal.pone.0223748 (PMC6793880; doi:10.1371/journal.pone.0223748)
Supplement: S1 File — (DOCX) [file pone.0223748.s001.docx]

# **Supplementary information 1**

**S1 Figure A. Kelp forest canopy cover quantified across areas surveyed.** *Nereocystis luetkeana* is the dominant canopy-forming species around *Haida Gwaii*, BC. Canopy cover was visually characterized on each transect as fragmented kelp cover (FKC), kelp cover (KC), and no kelp cover (NKC).

**S1 Figure B. Benthic reef habitat was quantified at the transect level across areas surveyed.** Substrate was classified categorically: gravel (G, 0.5 - 5cm), cobble (C, 5 cm – 30 cm), small boulder (SB30 cm – 1 m), medium boulder (MB, 1 m – 2 m), large boulder (LB, > 2 m), or bedrock reef (B, >10 m).

**S1 Table A.** Life-histories and foraging preferences of rocky reef fish. Maturity is indicated by the age at which 50% and/or the majority of females are reproductive. Data was compiled from multiple sources [1-8].

| **Species** | **Age of Maturity (yrs)** | **Max Age (yrs)** | **Home Range** | **Foraging Description** |
| --- | --- | --- | --- | --- |
| Black Rockfish  (*Sebastes melanops)* | 6-9 | 50 | 10 – 35 m | Generalists; mainly feed pelagic; mesopredator |
| Copper Rockfish  (*Sebastes caurinus)* | 6-7 | 50 | 2 - 33 m | Generalists; mainly feed benthic; mesopredator |
| China Rockfish  (*Sebastes nebulous)* | 6 | 79 | < 10 m | Generalists; mainly feed benthic; mesopredator |
| Quillback Rockfish  (*Sebastes maliger)* | 11 - 22 | 95 | < 10 m | Generalists; mainly benthic;  mesopredator |
| Yellowtail Rockfish  (*Sebastes flavidus)* | 6 | 64 | 22.5 km | Generalists; mainly feed pelagic; mesopredator |
| Canary Rockfish  (*Sebastes pinniger)* | 9-13 | 84 | 27 km | Generalists; mainly feed pelagic |
| Lingcod  (*Ophiodon elongatus)* | 4 | 25 | 2000 – 3000 m^2^ | Generalists; top predator |

**References**

1. Carlson HR, Haight RE. Evidence for a home site and homing of adult yellowtail rockfish, Sebastes flavidus. J Fish Res Board Can. 1972;29: 1011–1014.

2. Matthews KR. A telemetric study of the home ranges and homing routes of copper and quillback rockfishes on shallow rocky reefs. Can J Zoo. 1990;68: 2243–2250.

3. Eldridge MB, Whipple JA, Bowers MJ, Jarvis BM, Gold J. Reproductive performance of yellowtail rockfish, *Sebastes flavidus*. Environ Biol Fish. 1991;30: 91–102.

4. Love MS, Yoklavich M, Thorsteinson LK. The rockfishes of the northeast pacific. Univ of California Press; 2002.

5. Beaudreau AH, Essington TE. Spatial, temporal, and ontogenetic patterns of predation on rockfishes by lingcod. Trans Am Fish Soc. 2007;136: 1438–1452.

6. Parker SJ, Rankin PS, Olson JM, Hannah RW. Movement patterns of black rockfish (*Sebastes melanops*) in Oregon coastal waters. Biology, assessment, and management of North Pacific rockfishes. Juneau, AK: Alaska Sea Grant. 2007. pp. 39–57.

7. Tolimieri N, Andrews K, Williams G, Katz S, Levin PS. Home range size and patterns of space use by lingcod, copper rockfish and quillback rockfish in relation to diel and tidal cycles. Mar Ecol Prog Ser. 2009;380: 229–243.

8. Hannah RW, Rankin PS. Site fidelity and movement of eight species of pacific rockfish at a high-relief rocky reef on the Oregon coast. N Am J Fish Manag. 2011;31: 483–494.
