# Supplementary material for: Expanded consumer niche widths may signal an early response to spatial protection
Source: PLoS One. 2019 Oct 15;14(10):e0223748. doi: 10.1371/journal.pone.0223748 (PMC6793880; doi:10.1371/journal.pone.0223748)
Supplement: S2 File — (DOCX) [file pone.0223748.s002.docx]

# **Supplementary information 2**

**S2 Appendix A. Diet methods and results.**

(i) *Methods* - We enumerated, weighed (nearest mg), and identified prey found in rockfish stomachs to the lowest possible taxonomic level using a 50X power dissecting microscope. Samples too small to register a weight were assigned 0.0001 mg to be accounted for in analysis. Unidentifiable taxa were weighed, placed into an unknown category, and given a conservative total abundance of 1. Prey were grouped functionally (Appendix 3B). Prey composition was measured by abundance (%N), mass (% M), and frequency of occurrence (%O) for each rockfish species in each area (Eq. 1). Index of relative importance (%IRI) was also calculated as an unbiased measure in respect to prey size and to minimize variance (Pinkas et al. 1971, Eq. 2).

$$Equation 1 \%X_{i}= \frac{100* X_{i}}{\sum_{i=l}^{n} X_{i}}$$

$$Equation 2$$

Where, X is the total number (N), total mass (M), or occurrence (O) of prey *i* in a unit, respectively, and *n* is the total number of prey found in a unit (Equation 1). IRI was calculated as the percent index of relative importance of prey *i* in a unit to be comparable between areas (Equation 2). Prey richness was assessed by area as the total number of functional groups in all rockfish caught in a given area.

(ii) *Prey functional groups* - Main prey groupings and trophic levels of rockfish stomach contents. Contents were identified down to the lowest taxonomic group.

| **Functional Group** | **Taxa Included** |
| --- | --- |
| Amphipod | Amphipoda, Gammaridea |
| Bivalve | Cardiidae, *Acila castrensis, Clinocardium nuttallii, Musculus Musculus sp.* |
| Brittle star | Ophiuroidea |
| Bryozoans | Bryozoa |
| Caprellid | Caprellida |
| Cephalopod | Cephalopoda (beak), Octopoda, Teuthida |
| Chiton | Polyplacophora, radula, plates |
| Crabs | *Scrya acutifrons, Pugettia richii, Mimulus foliatus, Pugettia gracilis, Lophopanopaus bellus, Petrolisthes sp., Cancer productus, Cancer oregonensis, Cancer branneri, Cancer sp.* |
| Cucumber | Holothuroidea |
| Fish | *Sebastes flavidus,* Agonidae, Pholidae, Cottoidea, fish scales, fish bones |
| Gastropod | *Calliostoma ligatum, Astraea gibberosa, Granulina margaritula, Haliotis kamtschatkana, Calliostoma sp., Margarites sp., Olivella sp.,* Velutinidae*,* Gasrtopoda |
| Isopod | *Idotea wosnesenskii* |
| Oblelia | *Obelia sp.* |
| Pelagic Zooplankton | Mysidacea, Euphausiacea |
| Plants | *Phyllospadix sp., Zostera sp.,* Pine needles |
| Polycheate | Polychaeta, Aphrodita sp., Syllidae |
| Shrimp | Caridea, Dendrobranchiata |
| Urchin | *Strongylocentrotus purpuratus* |
| Unknown crustacean | Crustacean material and parts |
| Unknown | Unknown |

(iii) Stomach contents of rockfish caught in Fished South, the MPA, and Fished North, including species richness (total number of prey items) of prey in rockfish stomachs at the community level, and prey composition by % Mass and % Index of Relative Importance (IRI) of black, china, copper, and quillback rockfish species. Colours represent different prey groups.

***Prey Richness***


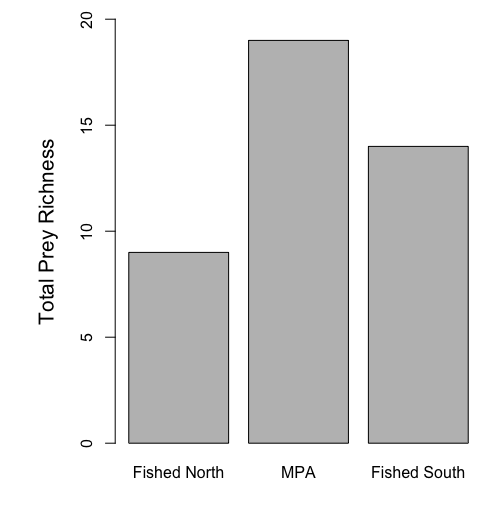


***Stomach Contents***

**S2 Table A. Summary of isotopic results: mean and standard deviation (SD) of δ^13^C and δ^15^N, and their lengths (cm), of rockfish collected at the Gwaii Haanas rocky reef areas.**

| **Species** | **Area** | **n** | **Length (cm)** | | **δ^13^C** | | **δ^15^N** | |
| --- | --- | --- | --- | --- | --- | --- | --- | --- |
|  |  |  | **Mean** | **SD** | **Mean** | **SD** | **Mean** | **SD** |
| Black Rockfish  (*S. melanops*) | Fished South | 5 | 75.4 | 13.0 | -18.44 | 0.41 | 14.42 | 0.25 |
|  | Fished North | 10 | 63.0 | 16.7 | -17.31 | 0.26 | 14.25 | 0.27 |
|  | MPA | 9 | 70.0 | 17.2 | -17.57 | 0.36 | 14.52 | 0.74 |
| Canary Rockfish (*S. pinniger)* | Fished North | 4 | 61.5 | 14.4 | -16.99 | 0.32 | 14.54 | 0.37 |
|  | MPA | 1 | 59.0 | - | -17.03 | - | 14.34 | - |
| China Rockfish (*S. nebulosus*) | Fished South | 3 | 51.7 | 12.5 | -16.33 | 0.26 | 15.23 | 0.36 |
|  | Fished North | 5 | 33.4 | 8.8 | -15.20 | 0.43 | 14.71 | 0.52 |
|  | MPA | 9 | 45.0 | 8.4 | -15.67 | 0.18 | 15.08 | 0.60 |
| Copper Rockfish (*S. caurinus*) | Fished South | 12 | 74.7 | 22.6 | -16.71 | 0.92 | 15.00 | 0.56 |
|  | Fished North | 2 | 32.0 | 12.7 | -16.22 | 0.63 | 15.26 | 0.19 |
|  | MPA | 12 | 68.9 | 24.8 | -15.67 | 0.34 | 15.69 | 0.46 |
| Quillback Rockfish (*S. maliger*) | Fished South | 11 | 58.5 | 7.70 | -18.07 | 0.52 | 14.80 | 0.24 |
|  | Fished North | 1 | 69.0 | - | -15.95 | - | 16.61 | - |
|  | MPA | 3 | 49.0 | 15.9 | -16.44 | 0.25 | 15.60 | 0.68 |
