# Supplementary material for: Expanded consumer niche widths may signal an early response to spatial protection
Source: PLoS One. 2019 Oct 15;14(10):e0223748. doi: 10.1371/journal.pone.0223748 (PMC6793880; doi:10.1371/journal.pone.0223748)
Supplement: S3 File — (DOCX) [file pone.0223748.s003.docx]

**Supplementary information 3**

**S3 Table A. Relative support of models explaining rockfish δ^15^N as a function of body length (cm) and area.** Models with parameters (K) were compared using likelihood of model given the data (Log(L)), K, and **∆**AIC_C_ values. Asterisks (*) represent an interaction term. Models shaded grey demonstrated fit similarity (<2 ∆ AIC_c_ among models) and were averaged to assess parameter effects (S3 Appendix 2).

| **Response** | **Model** | **K** | **AIC_c_** | **∆ AIC_c_** | **W_i_** | **Log(L)** |
| --- | --- | --- | --- | --- | --- | --- |
| Rockfish δ^15^N | Length + Area + Length*Area | 8 | 171.2 | 0 | 0.56 | -76.68 |
|  | Length + Area | 6 | 172.8 | 1.6 | 0.25 | -79.88 |
|  | Area | 5 | 174.46 | 3.26 | 0.11 | -81.86 |
|  | Length | 4 | 175.23 | 4.03 | 0.07 | -83.37 |
|  | Intercept | 3 | 178.82 | 7.62 | 0.01 | -86.27 |

**S3 Figure A. Scaled parameter estimates from model averaging of models predicting rockfish δ^15^N using rockfish length, area (MPA, Fished North, Fished South), and interactive effects (denoted by an asterisk) among the factors.** For an area effect, Fished South was used as the reference level in which estimates are relative to.

**S3 Figure B. Mean biomass and density (± SE) of reef fish observed on SCUBA transects (n = 4 transects per site) in 3 sites within Fished North, the MPA (grey), and Fished South.**

**S3 Table B. Model comparisons.** Evidence for alternative models examining the effects of protection status, kelp cover, benthic habitat, and depth on rockfish community and species-level (i) biomass (log) and (ii) density. Models with parameters (K) were compared using delta Akaike’s Information Criterion (∆AIC_c_) values, and Akaike weights (W_i_) representing weight of evidence for model *i*. Models include *site* as a random factor. The top 5 models for each response variable is shown. Well supported models (∆AIC_c_ <4) were averaged to determine the importance of individual factors. Models in bold had strong support (∆AIC_c_ < 2).

1. Biomass models

| **Response** | **Model** | **K** | **Log(L)** | **AIC_c_** | **∆ AIC_c_** | **W_i_** |
| --- | --- | --- | --- | --- | --- | --- |
| *Rockfish* | status + benthic habitat | 5 | -77.8 | 167.6 | 0.0 | 0.24 |
| *Community* | status + benthic habitat + kelp | 6 | -76.7 | 168.3 | 0.7 | 0.17 |
| *Biomass* | status + benthic habitat + kelp + depth | 7 | -75.3 | 168.7 | 1.0 | 0.14 |
|  | benthic habitat | 4 | -79.9 | 169.1 | 1.5 | 0.11 |
|  | status + benthic habitat + depth | 6 | -77.8 | 170.5 | 2.8 | 0.06 |
| *Black Rockfish* | status + benthic habitat + kelp | 6 | -60.7 | 136.4 | 0.0 | 0.42 |
| *Log Biomass* | status + benthic habitat | 5 | -63.0 | 138.1 | 1.7 | 0.18 |
|  | status + benthic habitat + kelp + depth | 7 | -60.5 | 139.0 | 2.7 | 0.11 |
|  | status + benthic habitat + depth | 6 | -62.7 | 140.2 | 3.8 | 0.06 |
|  | benthic habitat | 4 | -65.6 | 140.5 | 4.2 | 0.05 |
| *China Rockfish* | benthic habitat + depth | 5 | -77.0 | 166.1 | 0.0 | 0.18 |
| *Log Biomass* | kelp | 4 | -78.5 | 166.3 | 0.2 | 0.16 |
|  | benthic habitat + kelp | 5 | -77.2 | 166.3 | 0.3 | 0.16 |
|  | depth | 4 | -78.7 | 166.7 | 0.6 | 0.13 |
|  | kelp + depth | 5 | -77.9 | 167.7 | 1.6 | 0.08 |
| *Copper Rockfish* | intercept | 3 | -87.5 | 181.8 | 0.0 | 0.29 |
| *Log Biomass* | kelp | 4 | -86.9 | 183.0 | 1.2 | 0.16 |
|  | depth | 4 | -87.3 | 183.8 | 2.0 | 0.11 |
|  | status | 4 | -87.4 | 184.1 | 2.3 | 0.09 |
|  | rugosity | 4 | -87.5 | 184.3 | 2.5 | 0.09 |
| *Quillback Rockfish* | kelp + depth | 5 | -72.7 | 157.3 | 0.0 | 0.17 |
| *Log Biomass* | depth | 4 | -74.1 | 157.4 | 0.1 | 0.17 |
|  | rugosity + kelp + depth | 6 | -71.4 | 157.7 | 0.3 | 0.15 |
|  | rugosity + depth | 5 | -73.2 | 158.4 | 1.0 | 0.10 |
|  | intercept | 3 | -76.2 | 159.2 | 1.8 | 0.07 |
| *Lingcod* | kelp | 4 | -37.7 | 84.8 | 0.0 | 0.27 |
| *Log Biomass* | intercept | 3 | -39.6 | 85.9 | 1.2 | 0.15 |
|  | rugosity + kelp | 5 | -37.4 | 86.9 | 2.1 | 0.10 |
|  | status + kelp | 5 | -37.5 | 87.0 | 2.3 | 0.09 |
|  | kelp + depth | 5 | -37.6 | 87.2 | 2.4 | 0.08 |

Note: Not all models averaged are shown if more than 5 models have ∆AIC_c_ values < 4.

1. Density models

| **Response** | **Model** | **K** | **Log(L)** | **AIC_c_** | **∆ AIC_c_** | | **W_i_** |
| --- | --- | --- | --- | --- | --- | --- | --- |
| *Rockfish* | status + benthic habitat + kelp + depth | 6 | -74.9 | 164.6 | 0.0 | | 0.55 |
| *Community* | benthic habitat + kelp + depth | 5 | -76.7 | 165.3 | 0.7 | | 0.39 |
| *Density* | status + benthic habitat + kelp | 5 | -78.9 | 169.7 | 5.1 | | 0.04 |
|  | benthic habitat + kelp | 4 | -80.8 | 170.9 | 6.3 | | 0.02 |
|  | status + benthic habitat + depth | 5 | -96.0 | 203.9 | 39.3 | | 0.00 |
| *Black Rockfish* | **status + benthic habitat + kelp** | **5** | **-99.7** | **211.4** | **0.0** | | **0.58** |
| *Density* | status + benthic habitat + kelp + depth | 6 | -99.4 | 213.7 | 2.3 | | 0.19 |
|  | benthic habitat + kelp | 4 | -102.3 | 214.0 | 2.5 | | 0.16 |
|  | benthic habitat + kelp + depth | 5 | -101.9 | 215.8 | 4.4 | | 0.07 |
|  | status + benthic habitat + depth | 5 | -107.7 | 227.4 | 16.0 | | 0.00 |
| *China Rockfish* | **benthic habitat + depth** | **4** | **-21.9** | **53.1** | **0.0** | | **0.47** |
| *Density* | status + benthic habitat + depth | 5 | -21.8 | 55.5 | 2.4 | | 0.14 |
|  | benthic habitat + kelp + depth | 5 | -21.9 | 55.8 | 2.6 | | 0.13 |
|  | benthic habitat + kelp | 4 | -23.7 | 56.8 | 3.6 | | 0.08 |
|  | depth | 3 | -25.5 | 57.7 | 4.6 | | 0.05 |
| *Copper Rockfish* | kelp | 3 | -31.8 | 70.3 | 0.0 | | 0.28 |
| *Density* | depth | 3 | -32.2 | 71.2 | 0.9 | | 0.18 |
|  | kelp + depth | 4 | -31.5 | 72.2 | 1.9 | | 0.11 |
|  | benthic habitat + kelp | 4 | -31.6 | 72.5 | 2.1 | | 0.10 |
|  | status + kelp | 4 | -31.7 | 72.8 | 2.4 | | 0.08 |
| *Quillback Rockfish* | benthic habitat + kelp + depth | 5 | -18.3 | 48.6 | 0.0 | | 0.76 |
| *Density* | status + benthic habitat + kelp + depth | 6 | -18.0 | 51.0 | 2.4 | | 0.23 |
|  | kelp + depth | 4 | -24.9 | 59.0 | 10.4 | | 0.00 |
|  | status + kelp + depth | 5 | -24.2 | 60.4 | 11.8 | | 0.00 |
|  | benthic habitat | 3 | -28.2 | 63.1 | 14.5 | | 0.00 |
| *Lingcod* | intercept | 2 | -12.9 | 30.1 | 0.0 | | 0.16 |
| *Density* | kelp | 3 | -11.7 | 30.2 | 0.1 | | 0.16 |
|  | status + kelp | 4 | -10.6 | 30.4 | 0.3 | 0.14 | |
|  | status | 3 | -12.0 | 30.8 | 0.7 | 0.11 | |
|  | depth | 3 | -12.4 | 31.6 | 1.5 | 0.08 | |

Note: Not all models averaged are shown if more than 5 models have ∆AIC_c_ values < 4.

**S3 Figure C. Biomass model average estimates.** Scaled coefficients (circles) of model averaged estimates and unconditional 95% confidence intervals (lines) on rockfish biomass, based on supported models. If the confidence intervals bound 0, there is evidence of imprecision in the parameter coefficient and an effect is unsupported.

**
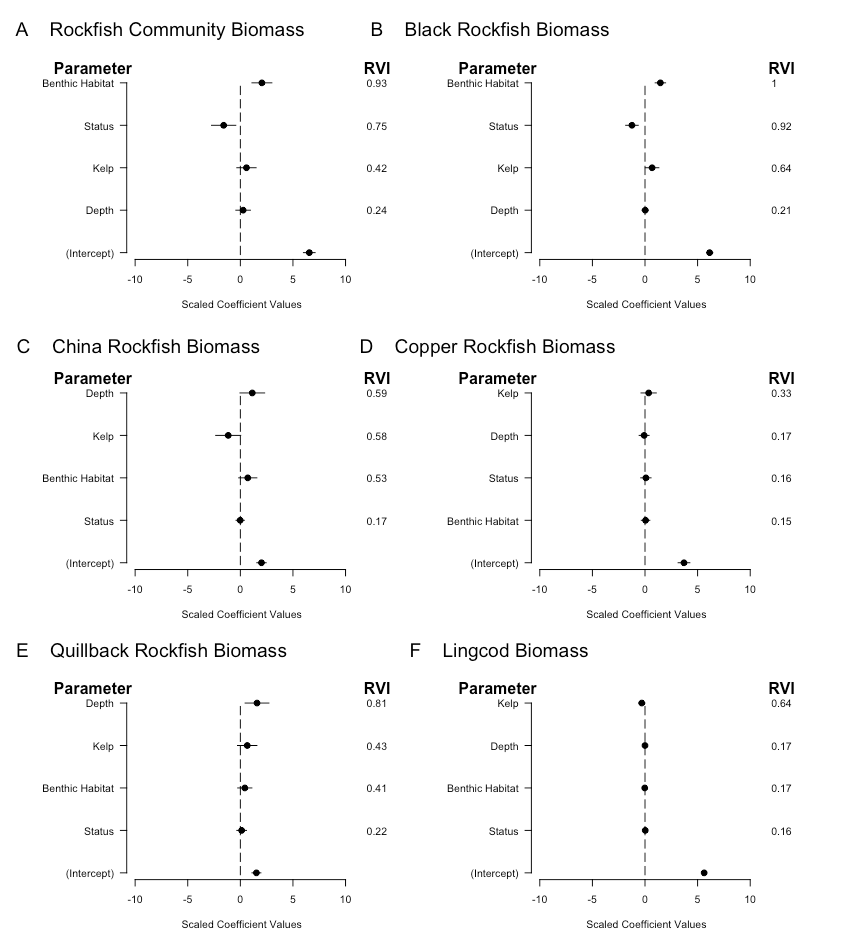
**

**S3 Figure D. Density model average estimates.** Scaled coefficients (circles) of model averaged estimates and unconditional 95% confidence intervals (lines) on rockfish densities across areas, based on supported models. If the confidence intervals bound 0, there is evidence of imprecision in the parameter coefficient and an effect is unsupported.

**
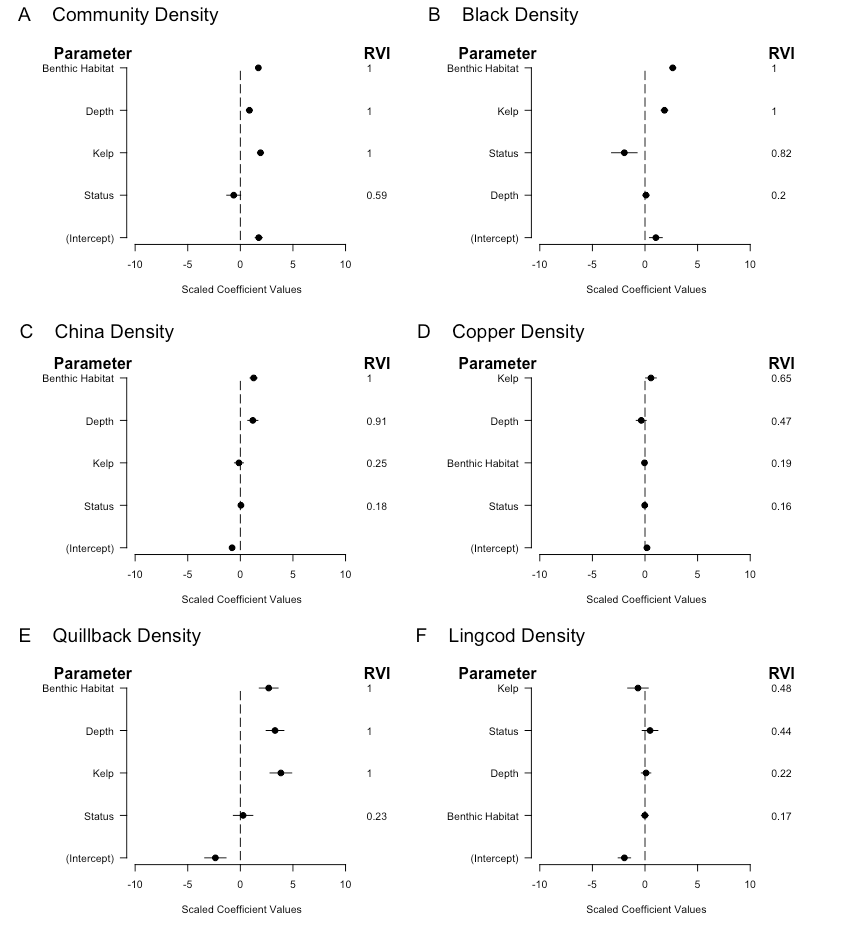
**

**S3 Figure E. Size frequency distributions of rocky reef fish observed by SCUBA**. (i) Community size-structure by area: Fished North (n = 216), MPA (n = 94), and Fished South (n = 76). (ii) Species-specific comparisons plotted by area. Histogram values indicate the percentage of fish at a given length bin, occurring in each area relative to all fish observed.


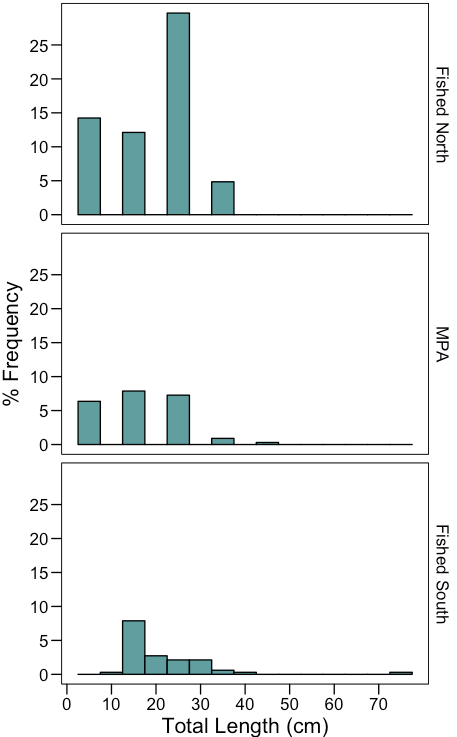


(i)

(ii)
